# Plug-and-Play Pairing via Defined Divalent Streptavidins^☆^

**DOI:** 10.1016/j.jmb.2013.09.016

**Published:** 2014-01-09

**Authors:** Michael Fairhead, Denis Krndija, Ed D. Lowe, Mark Howarth

**Affiliations:** Department of Biochemistry, University of Oxford, South Parks Road, Oxford OX1 3QU, UK

**Keywords:** LDLR, low-density lipoprotein receptor, MPD, 2-methyl-2,4-pentanediol, PDB, Protein Data Bank, PBS, phosphate-buffered saline, avidin, protein design, bivalent, supramolecular, nanotechnology

## Abstract

Streptavidin is one of the most important hubs for molecular biology, either multimerizing biomolecules, bridging one molecule to another, or anchoring to a biotinylated surface/nanoparticle. Streptavidin has the advantage of rapid ultra-stable binding to biotin. However, the ability of streptavidin to bind four biotinylated molecules in a heterogeneous manner is often limiting. Here, we present an efficient approach to isolate streptavidin tetramers with two biotin-binding sites in a precise arrangement, *cis* or *trans*. We genetically modified specific subunits with negatively charged tags, refolded a mixture of monomers, and used ion-exchange chromatography to resolve tetramers according to the number and orientation of tags. We solved the crystal structures of *cis*-divalent streptavidin to 1.4 Å resolution and *trans*-divalent streptavidin to 1.6 Å resolution, validating the isolation strategy and explaining the behavior of the Dead streptavidin variant. *cis*- and *trans*-divalent streptavidins retained tetravalent streptavidin's high thermostability and low off-rate. These defined divalent streptavidins enabled us to uncover how streptavidin binding depends on the nature of the biotin ligand. Biotinylated DNA showed strong negative cooperativity of binding to *cis*-divalent but not *trans*-divalent streptavidin. A small biotinylated protein bound readily to *cis* and *trans* binding sites. We also solved the structure of *trans*-divalent streptavidin bound to biotin-4-fluorescein, showing how one ligand obstructs binding to an adjacent biotin-binding site. Using a hexaglutamate tag proved a more powerful way to isolate monovalent streptavidin, for ultra-stable labeling without undesired clustering. These forms of streptavidin allow this key hub to be used with a new level of precision, for homogeneous molecular assembly.

## Highlights

• Streptavidin is a widely used nanohub: often, tetravalency makes assembly imprecise.
• We isolated divalent streptavidins with biotin-binding sites in *cis* or in *trans*.
• *cis*- and *trans*-divalent streptavidins retain exceptional biotin binding and thermostability.
• Binding large biotinylated ligands to adjacent binding sites is greatly disfavored.
• *cis*- and *trans*-divalent streptavidins represent a simple way to bridge biomolecules precisely.

## Graphical Abstract

**Figure:**
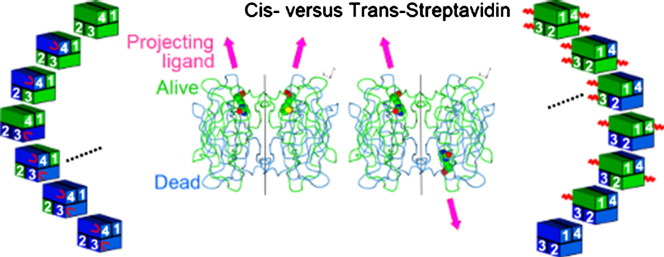

## Introduction

Streptavidin is a tetrameric protein from *Streptomyces avidinii* [1] that, along with the structurally similar protein from chicken egg white, avidin, exhibits extraordinarily high affinity for the vitamin biotin [2,3]. The femtomolar affinity of streptavidin/avidin for biotinylated targets, combined with resilience to harsh conditions and an on-rate of ~ 10^7^ M^− 1^ s^− 1^, is the basis for biotin's central role as a specific biological “glue” in diverse research methods and biotechnologies [1–3].

Although streptavidin has a long history of use for *capture* of biotinylated ligands from complex mixtures, it has gained many recent applications for precise and stable *assembly* in the emerging field of bionanotechnology [4–6]. Such assemblies can be centered on a single streptavidin, for example, streptavidin:MHC (major histocompatibility complex) tetramers, to analyze immune function [7]. Assemblies can also depend on multiple streptavidin molecules holding together networks, such as for amplification in histochemistry [8] or to bridge biomolecules to facilitate structure determination by electron microscopy, atomic force microscopy, and X-ray crystallography [9–11].

A central issue that needs addressing is to control the nature of the four binding sites in the streptavidin tetramer. While the binding of four ligands may be desirable in some applications, in other applications, it may be necessary to limit binding to only one, two, or three subunits to achieve the desired assemblies and geometries [11–13]. Splitting up the streptavidin tetramer into monomers or dimers is possible, but this has always been accompanied by orders of magnitude lower affinity for biotin [14–17], because of the contribution of the neighboring subunit to the biotin-binding site [1,18]. Such monomers or dimers give desirable reversibility for purification but forfeit much of the advantage of streptavidin's stability for assembly applications.

**Fig. 1:**
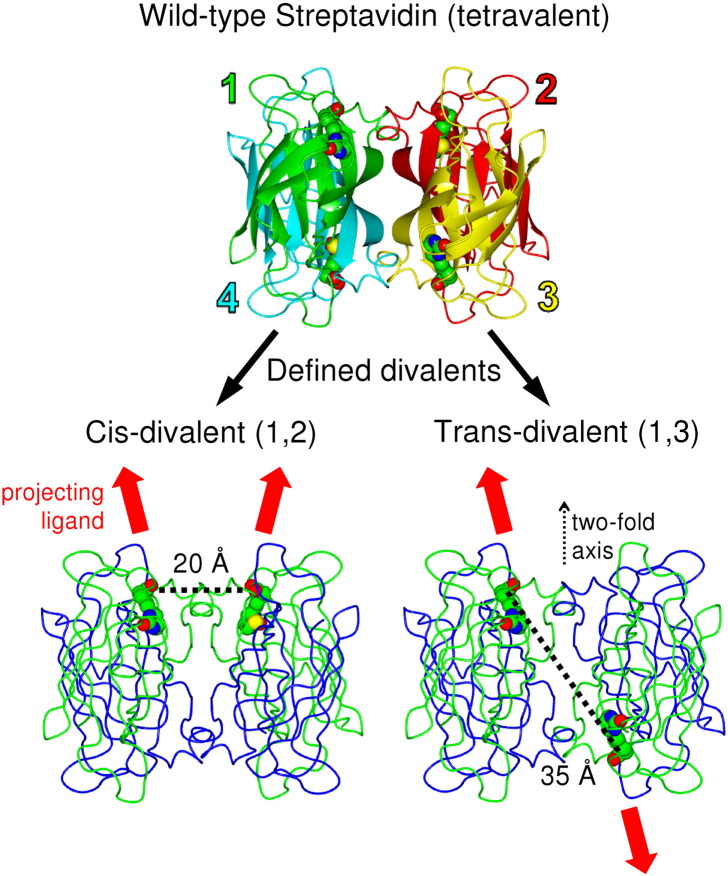
Principle of defined divalent streptavidins. Numbering of subunits in the streptavidin tetramer, with each monomer colored separately and biotin in space-fill (from PDB ID: 3RY2). Below are arrangements of 1,2 or 1,3 biotin-binding sites (biotin-bound monomers, green; non-binding monomers, blue), showing relative orientations and distances between the biotin carboxyl group carbons.

We previously generated streptavidin heterotetramers with exactly one, two, or three functional biotin-binding sites, through purifying tetramers containing defined numbers of non-biotin-binding “Dead” and His_6_-tagged wild-type subunits [19]. However, tetramers with two functional and two Dead subunits can assemble in *three* different arrangements. These arrangements are the 1,2, 1,3, and 1,4 divalent forms of streptavidin (Fig. 1). In the 1,2 arrangement the biotin-binding sites of streptavidin are in a *cis* configuration as the binding sites both point in the same direction (Fig. 1). However, in the 1,3 arrangement, both sites are in a *trans* configuration, pointing away from each other (Fig. 1). Taking a vector from N1 to the terminal carbon atom of biotin, in 1,2, both biotins point 23° from the two-fold axis in Fig. 1, while in 1,3, one biotin is 23° from this axis and the other is 160°. In 1,2, the terminal carbon atoms of the two biotins are 20 Å apart, and in 1,3, they are 35 Å apart (Fig. 1). Therefore, any biotinylated ligands to divalent streptavidin will adopt different distances and angles with respect to each other. Such mixed divalent species, containing all three arrangements, would not be suitable for applications where defined spatial control is necessary. For example, a *cis* arrangement may be suitable for capping biotinylated protein oligomers [20], resulting in molecules with a uniform length, while the *trans* arrangement may serve as a linker in the middle of such an assembly [20,21].

Control over subunit orientation could be achieved by generating single-chain dimers or tetramers [22], but the symmetry and folding characteristics of streptavidin mean that connection of subunits (using circular permutation) has been accompanied by impaired folding and affinity [16,23].

To generate a controlled and stable bridge for bionanotechnology, we demonstrate here a method for the simple isolation of streptavidin variants with precise *cis*-divalent or 1,3 *trans*-divalent arrangements. We confirmed the variant structures by crystallography and investigated the binding characteristics of these defined divalents, which helps to resolve decades of data about interaction between binding sites on streptavidin/avidin [24–28].

## Results

### Streptavidins with defined valencies and subunit orientation by engineering of charge

**Fig. 2:**
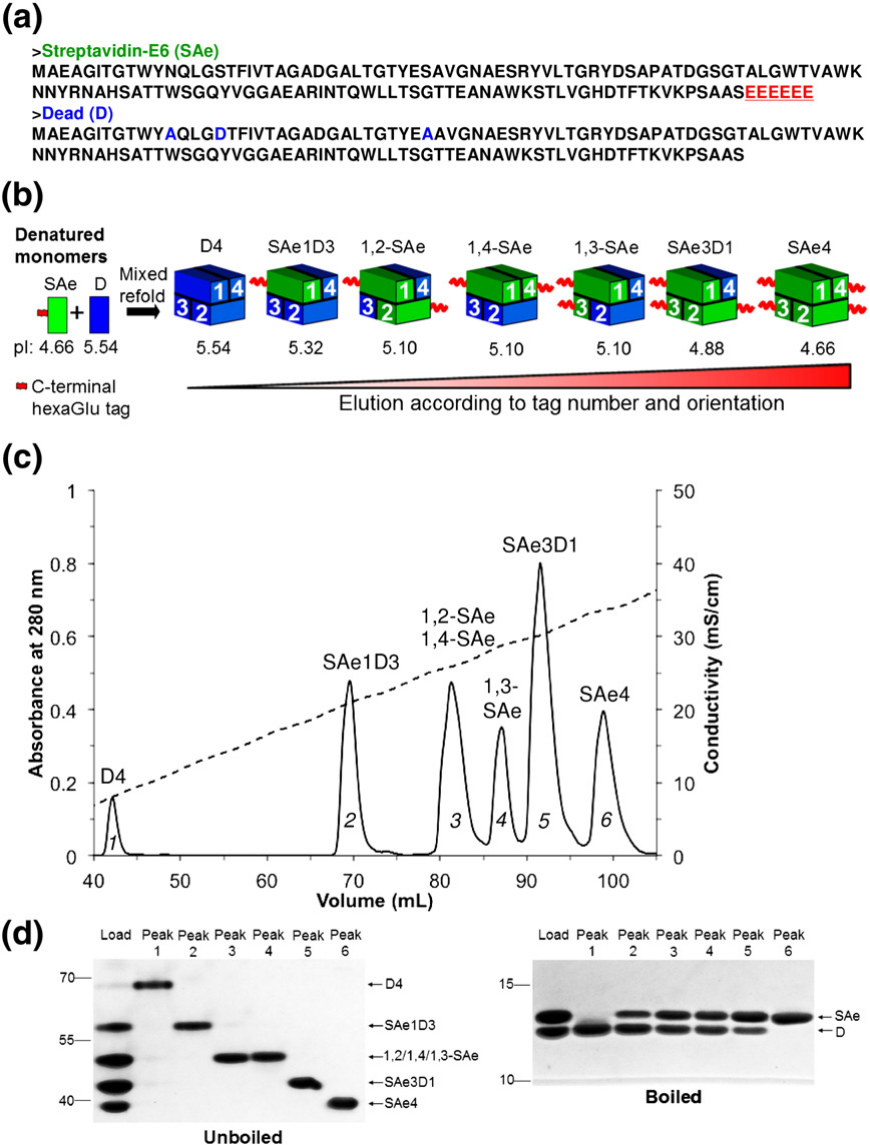
Generation of the 1,3 *trans*-divalent streptavidin. (a) Amino acid sequence of streptavidin-E_6_ (SAe) with E_6_ tag red and underlined, along with Dead (D) streptavidin showing residues impairing biotin binding in blue. (b) Generation of the different valency forms of SAe/D by mixed refold and ion exchange, with predicted p*I* of monomer and tetramer indicated. (c) Ion-exchange chromatogram of a mixture of tetramers containing different proportions of SAe and D. (d) Analysis of peaks from ion exchange by SDS-PAGE with Coomassie staining, unboiled (left) to show tetramer mobility, or boiled (right) to show subunit composition.

**Fig. 3:**
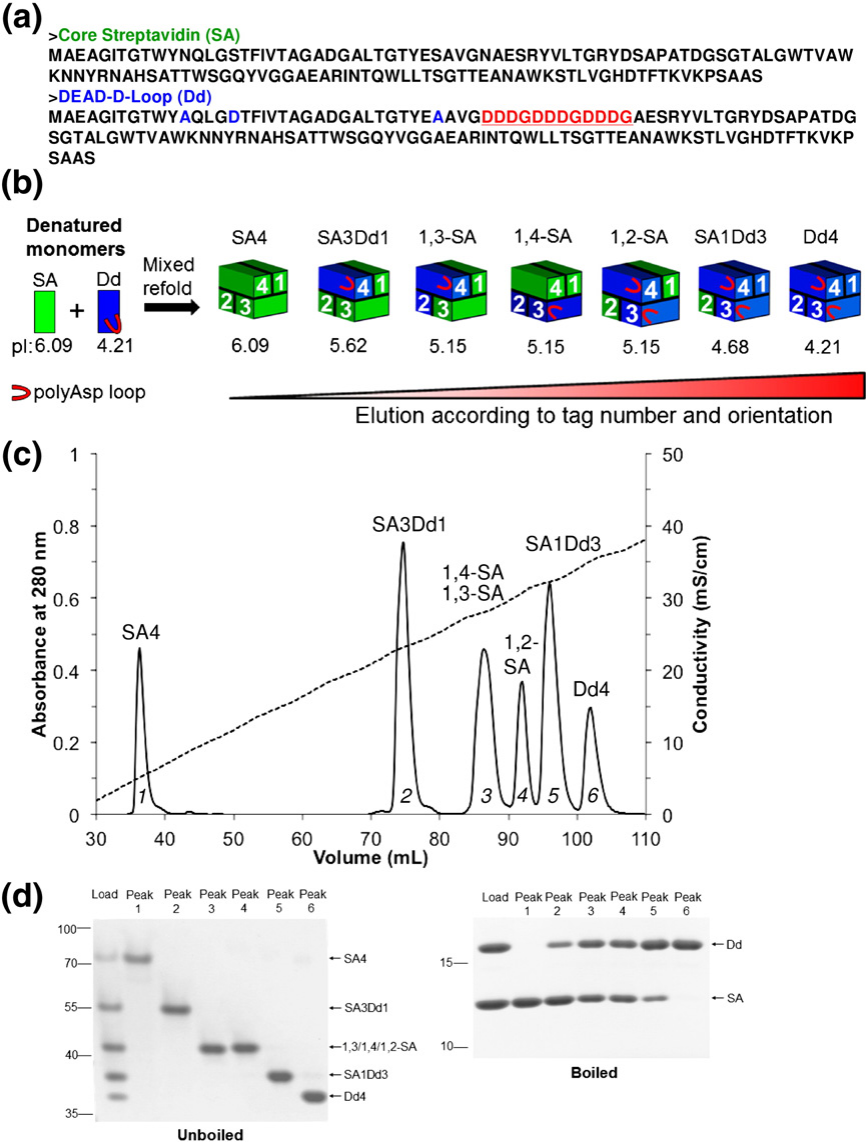
Generation of *cis*-divalent streptavidin. (a) Amino acid sequence of core streptavidin (SA), along with Dead (Dd) containing a polyaspartate insertion in the 3/4 loop (red and underlined) and residues impairing biotin binding in blue. (b) Generation of the different valency forms of SA/Dd by mixed refold and ion exchange, with predicted p*I* of monomer and tetramer indicated. (c) Ion-exchange chromatogram of a mixture of tetramers containing different proportions of SA and Dd. (d) Analysis of peaks from ion exchange by SDS-PAGE with Coomassie staining, unboiled (left) to show tetramer mobility, or boiled (right) to show subunit composition.

To prepare streptavidin tetramers with controlled pairs of biotin-binding sites (Fig. 1), we added charged tags at specific sites in the subunits to enable resolution of the tetramers by ion-exchange chromatography (Figs. 2 and 3). We refolded from inclusion bodies the mixture of charged and non-charged streptavidin variants and then separated the tetramers on a MonoQ column (anion exchange), since the tetramers with increasing negative charge required higher ionic strength for elution. Geometrically distinct species bearing the same number of charged tags can be separated from each other. On that basis, two different engineering strategies made it possible to isolate the *cis*-divalent and 1,3 *trans*-divalent forms (Fig. 1).

### C-terminal polyglutamate tag allowed 1,3 *trans*-divalent isolation

In the first instance, we placed a hexaglutamate tag at the C-terminus of core streptavidin monomer (termed SAe) (Fig. 2a). First, these charges ought to allow easy isolation of forms containing 0–4 biotin-binding sites by ion-exchange chromatography, giving clearer separation than the previous method of separation using a C-terminal His_6_ tag and Ni-NTA, where peaks from different tetramer forms largely overlapped [19,29]. Second the C-termini are close together between subunits 1 and 3 (27 Å) but far apart between subunits 1 and 2 (57 Å) or subunits 1 and 4 (50 Å), so that elution from the ion-exchange resin would be predicted to occur later for the 1,3 divalent (Fig. 1).

SAe was refolded together with Dead (D) streptavidin (N23A, S27D, S45A) [19] (Fig. 2a). Seven different tetramer forms are possible: one nullivalent, one monovalent, one trivalent, one tetravalent, and *three* divalent (Fig. 2b). The ion-exchange chromatogram showed six peaks (Fig. 2c), consistent with our hypothesis that 1,2 and 1,4 divalents would co-elute, while the 1,3 divalent would elute later.

We analyzed the eluted peaks by SDS-PAGE, where streptavidin will remain a tetramer without boiling (Fig. 2d) [30]. The tetramers do not run according to their molecular weight: the increasing negative charge increases migration of tetramers containing a higher proportion of SAe. Upon boiling in SDS, the tetramer breaks up into monomers and the relative proportion of SAe and D subunits is shown (Fig. 2d). This gel analysis confirmed that peak 1 was D4, peak 2 was the monovalent SAe1D3, peak 5 was the trivalent SAe3D1, peak 6 was the tetravalent SAe4, and peaks 3 and 4 were both divalent. A titration with biotin-4-fluorescein (whose fluorescence is quenched dramatically upon streptavidin binding) [31,32] confirmed the expected number of biotin-binding sites for D4, SAe4, SAe1D3, and the putative 1,3 *trans*-divalent (Supplementary Data, Fig. S1a).

We analyzed the eluted peaks by SDS-PAGE, where streptavidin will remain a tetramer without boiling (Fig. 2d) [30]. The tetramers do not run according to their molecular weight: the increasing negative charge increases migration of tetramers containing a higher proportion of SAe. Upon boiling in SDS, the tetramer breaks up into monomers and the relative proportion of SAe and D subunits is shown (Fig. 2d). This gel analysis confirmed that peak 1 was D4, peak 2 was the monovalent SAe1D3, peak 5 was the trivalent SAe3D1, peak 6 was the tetravalent SAe4, and peaks 3 and 4 were both divalent. A titration with biotin-4-fluorescein (whose fluorescence is quenched dramatically upon streptavidin binding) [31,32] confirmed the expected number of biotin-binding sites for D4, SAe4, SAe1D3, and the putative 1,3 *trans*-divalent (Supplementary Data Fig. S1a).

### Multiple aspartates in a surface loop allowed *cis*-divalent isolation

To isolate *cis*-divalent streptavidin (1,2 arrangement of biotin-binding subunits) (Fig. 1), we introduced a series of aspartates into the loop between β-strands 3 and 4 (L3,4) of the Dead subunit (Fig. 3a). After exploring a number of different insertions for optimal expression and separation, we chose the sequence termed Dd (Fig. 3a). Introduction of the charges in this position means that in the 1,3 and 1,4 arrangements, these charged regions should be far apart, while in the 1,2 arrangement, the charged loops should be apposed (Fig. 3b). Since L3,4 is important for biotin binding affinity [1,18,33], it was necessary to insert these residues on the Dead subunit, so that the *cis*-divalent streptavidin could maintain high biotin binding affinity. Ion-exchange chromatography of a mixed refold of SA/Dd again allowed efficient separation of tetramers, giving six peaks, two of which corresponded to divalent species (Fig. 3b and c).

We analyzed peaks by SDS-PAGE with and without boiling. For unboiled samples, the negative charge on Dd accelerated the mobility of the tetramers (Fig. 3d). Upon boiling, the varying intensities of SA and Dd bands clearly showed that the tetramers contained the expected ratios of each subunit (Fig. 3d). This gel analysis confirmed that peak 1 was tetravalent SA4, peak 2 was the trivalent SA3Dd1, peak 5 was the monovalent SA1Dd3, peak 6 was the non-binding Dd4, and peaks 3 and 4 were both divalent. A titration of the different tetramer forms with biotin-4-fluorescein also confirmed the expected number of biotin-binding sites (Supplementary Data Fig. S1b). Overall, the introduction of a series of negative charges into the surface-exposed 3,4-loop of streptavidin allowed the isolation of a second divalent species, *cis*-divalent.

We analyzed peaks by SDS-PAGE with and without boiling. For unboiled samples, the negative charge on Dd accelerated the mobility of the tetramers (Fig. 3d). Upon boiling, the varying intensities of SA and Dd bands clearly showed that the tetramers contained the expected ratios of each subunit (Fig. 3d). This gel analysis confirmed that peak 1 was tetravalent SA4, peak 2 was the trivalent SA3Dd1, peak 5 was the monovalent SA1Dd3, peak 6 was the non-binding Dd4, and peaks 3 and 4 were both divalent. A titration of the different tetramer forms with biotin-4-fluorescein also confirmed the expected number of biotin-binding sites (Supplementary Data Fig. S1b). Overall, the introduction of a series of negative charges into the surface-exposed 3,4-loop of streptavidin allowed the isolation of a second divalent species, *cis*-divalent.

### Crystal structures of *cis*-divalent and 1,3 *trans*-divalent streptavidins

**Fig. 4:**
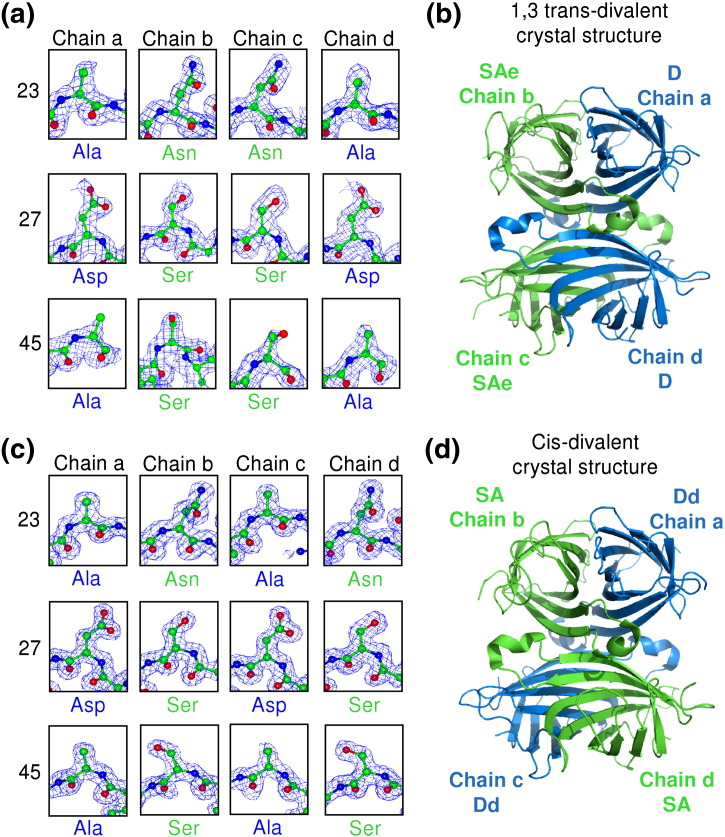
Crystal structures of divalent streptavidins. (a) Electron density (2*F*_o_ − *F*_c_ contoured at 1 rmsd) at residues 23, 27, and 45 in the 1,3 *trans*-divalent structure shown for chains b/c (SAe, green) and chains a/d (D, blue), with the side chain found indicated below. (b) Crystal structure of the 1,3 *trans*-divalent streptavidin, with green showing the biotin-binding SAe subunits and blue showing the non-biotin-binding D subunits. (c) Electron density in the *cis*-divalent structure, with chains b/d (SA, green) and chains a/c (Dd, blue). (d) Crystal structure of *cis*-divalent streptavidin, with green showing the biotin-binding SA subunits and blue showing the non-biotin-binding Dd subunits.

To confirm these divalent arrangements suggested by the ion-exchange elution profiles, we solved at high resolution the crystal structures of the 1,3 *trans*- and *cis*-divalent streptavidin (Fig. 4). Initially, all our crystal forms only contained two subunits in the asymmetric unit, which did not allow us to distinguish the possible divalent forms. However, one condition obtained using the Morpheus crystal screen [34] yielded the desired asymmetric unit for the *cis*-divalent streptavidin containing all four subunits. For the 1,3 *trans*-divalent form, crystallization with 2-methyl-2,4-pentanediol (MPD) gave us the desired asymmetric unit [35]. The obtained 1,3 *trans*-divalent crystal structure was solved at 1.6 Å resolution (Fig. 4b and Table 1), and *cis*-divalent streptavidin was solved at 1.4 Å resolution (Fig. 4d and Table 1), with the phases in each case solved using molecular replacement.

We identified each subunit (whether SAe/D or SA/Dd) unambiguously by examining whether the electron density around residues 23, 27, and 45 corresponded to the residues of the wild-type or Dead subunit (N23A, S27D, S45A). For the putative 1,3 *trans*-divalent, the electron density closely fitted residues 23, 27, and 45 only if chains b/c were SAe and chains a/d were D (Fig. 4a), confirming that the biotin-binding subunits indeed had a 1,3 *trans* orientation (Fig. 4b). For the putative *cis*-divalent sample, the electron density closely fits if chains a/c are Dd and chains b/d are SA (Fig. 4c), also confirming that the biotin-binding subunits indeed had a *cis* orientation (Fig. 4d).

Furthermore, while the electron density around L3,4 could be clearly observed in the SA chains b and d of the *cis*-divalent form, the electron density of L3/4 was poorly defined in Dd chains a and c (Supplementary Data Fig. S2). The engineered L3,4 of Dd (chains a/c) is expected to be highly flexible, which would lead to weak diffraction in this region.

Furthermore, while the electron density around L3,4 could be clearly observed in the SA chains b and d of the *cis*-divalent form, the electron density of L3/4 was poorly defined in Dd chains a and c (Supplementary Data Fig. S2). The engineered L3,4 of Dd (chains a/c) is expected to be highly flexible, which would lead to weak diffraction in this region.

**Fig. 5:**
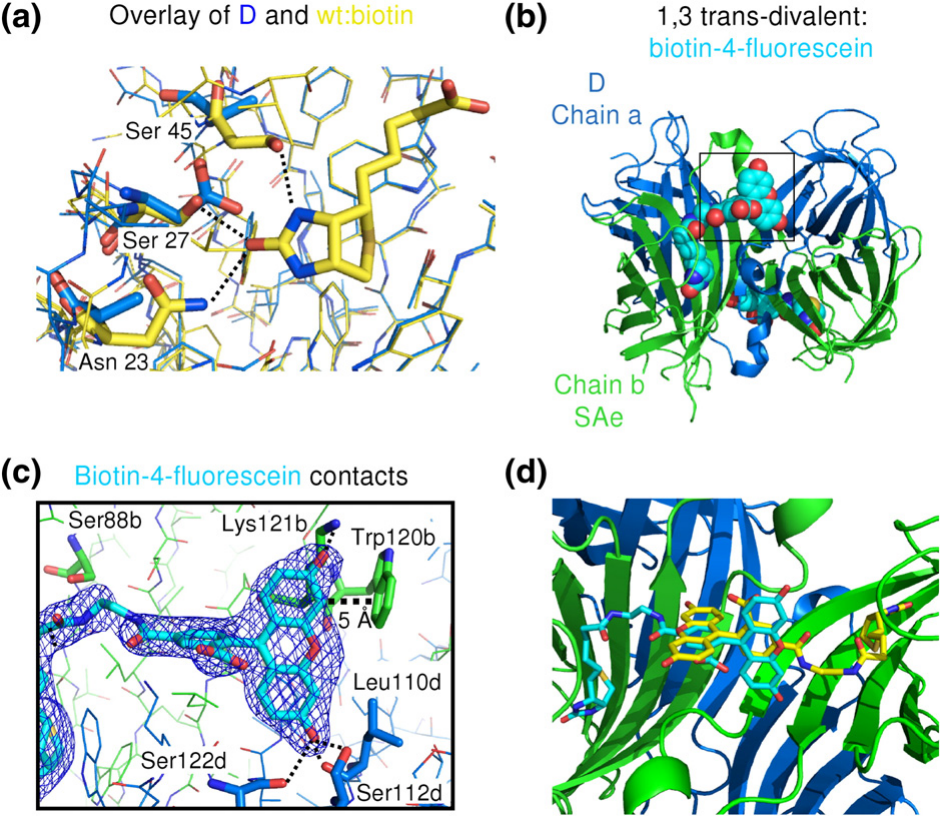
Structures of streptavidin interaction with ligands. (a) Structure of the biotin-binding pocket in the Dead subunit (N23A, S27D, and S45A), showing chain a of the 1,3 *trans*-divalent streptavidin (carbon atoms in blue) overlaid with biotin-bound wild-type streptavidin (PDB ID: 3RY2, carbon atoms in yellow). Hydrogen bonds from residues 23, 27, and 45 to biotin in wild-type streptavidin are shown as broken lines. (b) Crystal structure of the 1,3 *trans*-divalent streptavidin bound to biotin-4-fluorescein (space-fill, carbons in cyan), with green for SAe and blue for D subunits. (c) Residues surrounding the fluorescein tail (carbon atoms in cyan) in the 1,3 *trans*-divalent:biotin-4-fluorescein structure, labeled according to chain (b, carbon atoms in green; d, carbon atoms in blue), and with putative interactions as broken lines. Electron density for biotin-4-fluorescein is shown as blue mesh and contoured at 1 rmsd. (d) Clash for *cis*-bound biotin-4-fluorescein, generated from the 1,3 *trans*-divalent biotin-4-fluorescein structure, if two biotin-4-fluorescein molecules (carbon atoms of one in yellow and the other in cyan) were to bind in the same conformation in *cis* as they do in 1,3 *trans*-divalent.

The rmsd of any of the chains in 1,3 *trans*-divalent and *cis*-divalent streptavidin is less than 0.5 Å to chain a of the 0.95-Å structure of streptavidin [Protein Data Bank (PDB) ID: 3RY2] [35]. The Dead streptavidin subunit has not been previously analyzed by crystallography, and the changes to the structure are localized to the mutated residues, consistent with the comparable thermostability of tetramers containing wild-type or Dead subunits [19,33]. The overlay of D and a wild-type subunit bound to biotin illustrates how the three mutations in D greatly reduced the number of polar contacts to biotin (Fig. 5a). In the overlay, Asp27 has van der Waals clash with biotin and there would be repulsion between the Asp27 carboxylate and biotin's carbonyl oxygen lone pair (Fig. 5a).

### Crystal structure of biotin-4-fluorescein bound to streptavidin

To understand interaction between ligand binding sites, we solved 1,3 *trans*-divalent streptavidin's crystal structure bound to biotin-4-fluorescein. Biotin-4-fluorescein represents the largest ligand where there is a crystal structure with streptavidin or avidin, as well as a widely used probe of the binding kinetics and capacity of biotin-binding proteins [19,31,32,36–38]. The fluorescence of biotin-4-fluorescein is quenched ~ 90% on binding to streptavidin/avidin, allowing a clear and sensitive test of ligand binding without any separation step [31,32]. Our 2.3-Å-resolution structure shows the 1,4 dimer in the asymmetric unit (one copy of SAe and one copy of D). Building the native streptavidin tetramer from this dimer shows that the two biotin-4-fluorescein molecules are in the expected *trans* (1,3) arrangement (Fig. 5b). All the contacts of the 1,3 *trans*-divalent streptavidin with the biotin moiety are as expected [18]. Fluorescein and the ethylene diamine spacer form several non-polar contacts to streptavidin not observed with biotin (Fig. 5c). Features that have been proposed to quench fluorescein are photoinduced electron transfer to nearby tryptophans or tyrosines [39], co-planarity of the xanthenone and benzoate rings [40], and hydrogen bonding to O3 of fluorescein [41]. Trp120 is 5 Å from fluorescein (Fig. 5c) and may explain part of the quenching of fluorescence.

Biotin-4-fluorescein bound in chain b reaches over and interacts with residues of chain d. However, we do not believe this conformation of biotin-4-fluorescein is the same as when four ligands are bound to the same tetramer: if the SAe:biotin-4-fluorescein complex were in a *cis* arrangement, there would be an obvious clash between the fluorescein moieties of neighboring ligands (Fig. 5d).

### Low off-rate and high thermostability of defined divalents

**Fig. 6:**
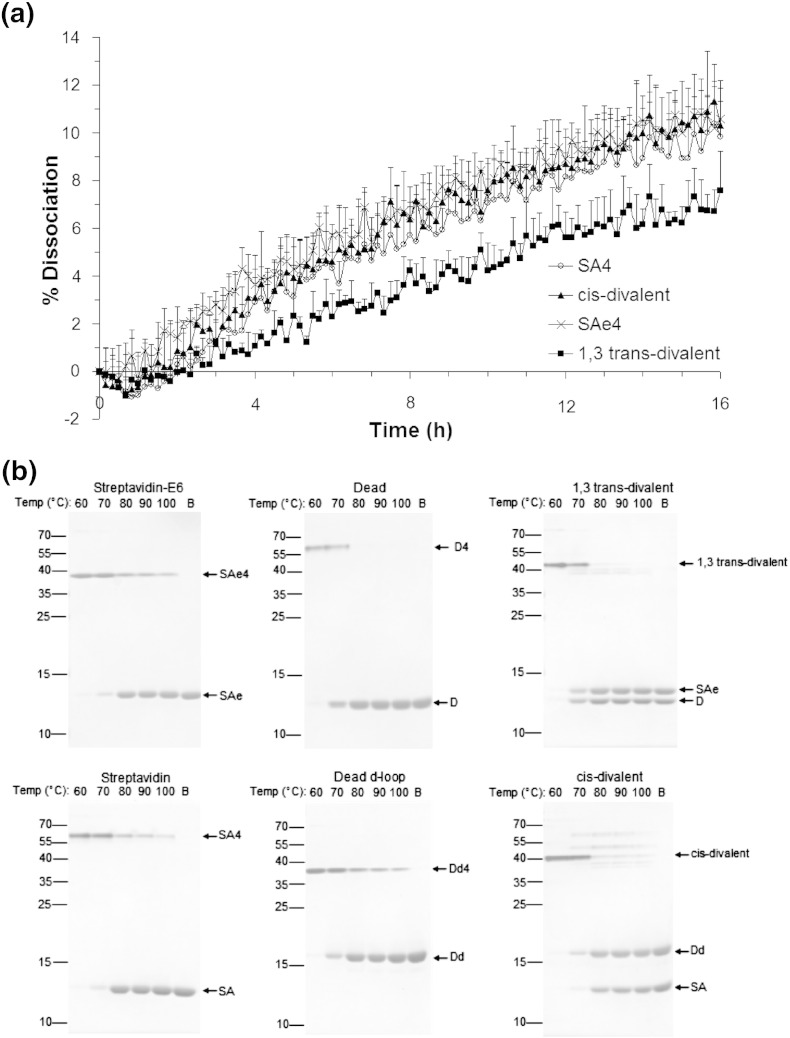
Off-rate and thermostability of divalent streptavidins. (a) Biotin-4-fluorescein dissociation over time at 37 °C in the presence of competing biotin, for a tetramer of wild-type streptavidin subunits (SA4), a tetramer of SAe subunits (SAe4), and *cis*-divalent or 1,3 *trans*-divalent streptavidin (mean of triplicate + 1 SD). (b) Tetramer thermostability for streptavidin variants determined by SDS-PAGE with Coomassie staining, following incubation in PBS for 3 min at the indicated temperatures. In B, the protein was boiled in the presence of SDS to achieve complete conversion to monomers. The mobilities of the starting tetramer and the resulting monomers are marked.

Having confirmed the identity of the defined divalents, we initially assessed these proteins by measuring the off-rate for a biotin conjugate (Fig. 6a). The strong biotin-binding stability of wild-type streptavidin was retained by the defined divalents. Both divalents had dissociation rates for biotin-4-fluorescein comparable to the original SA4 or SAe4 tetramers. In fact, the binding stability for the 1,3 *trans*-divalent streptavidin was slightly superior to that of the *cis*-divalent one (*P* ≤ 0.001, *n* = 3) and that of the wild-type SA4 tetramer (*P* ≤ 0.001, *n* = 3, one-way ANOVA with Tukey's multiple comparison) (Fig. 6a).

We next compared the thermostability of the two divalent forms with tetramers containing only the constituent monomers. We incubated at varying temperatures and then analyzed the resulting protein forms by SDS-PAGE (Fig. 6b). Both *cis*-divalent and 1,3 *trans*-divalent forms started to denature to their constituent monomers above 70 °C, in a similar fashion to SA4, Dd4, SAe4, and D4. Some mixing of the divalent tetramers into new species could be observed at 70 °C before full denaturation into the monomer species (Fig. 6b). To check whether this mixing would be an issue at 37 °C or room temperature, we incubated *cis*-divalent and 1,3 *trans*-divalent streptavidins for 1–8 days, but we observed no detectable dissociation to rearranged tetramers or to constituent monomers (Supplementary Data, Fig. S3).

We next compared the thermostability of the two divalent forms with tetramers containing only the constituent monomers. We incubated at varying temperatures and then analyzed the resulting protein forms by SDS-PAGE (Fig. 6b). Both *cis*-divalent and 1,3 *trans*-divalent forms started to denature to their constituent monomers above 70 °C, in a similar fashion to SA4, Dd4, SAe4, and D4. Some mixing of the divalent tetramers into new species could be observed at 70 °C before full denaturation into the monomer species (Fig. 6b). To check whether this mixing would be an issue at 37 °C or room temperature, we incubated *cis*-divalent and 1,3 *trans*-divalent streptavidins for 1–8 days, but we observed no detectable dissociation to rearranged tetramers or to constituent monomers (Supplementary Data Fig. S3).

### Binding of large biotinylated ligands by defined divalent streptavidins

**Fig. 7:**
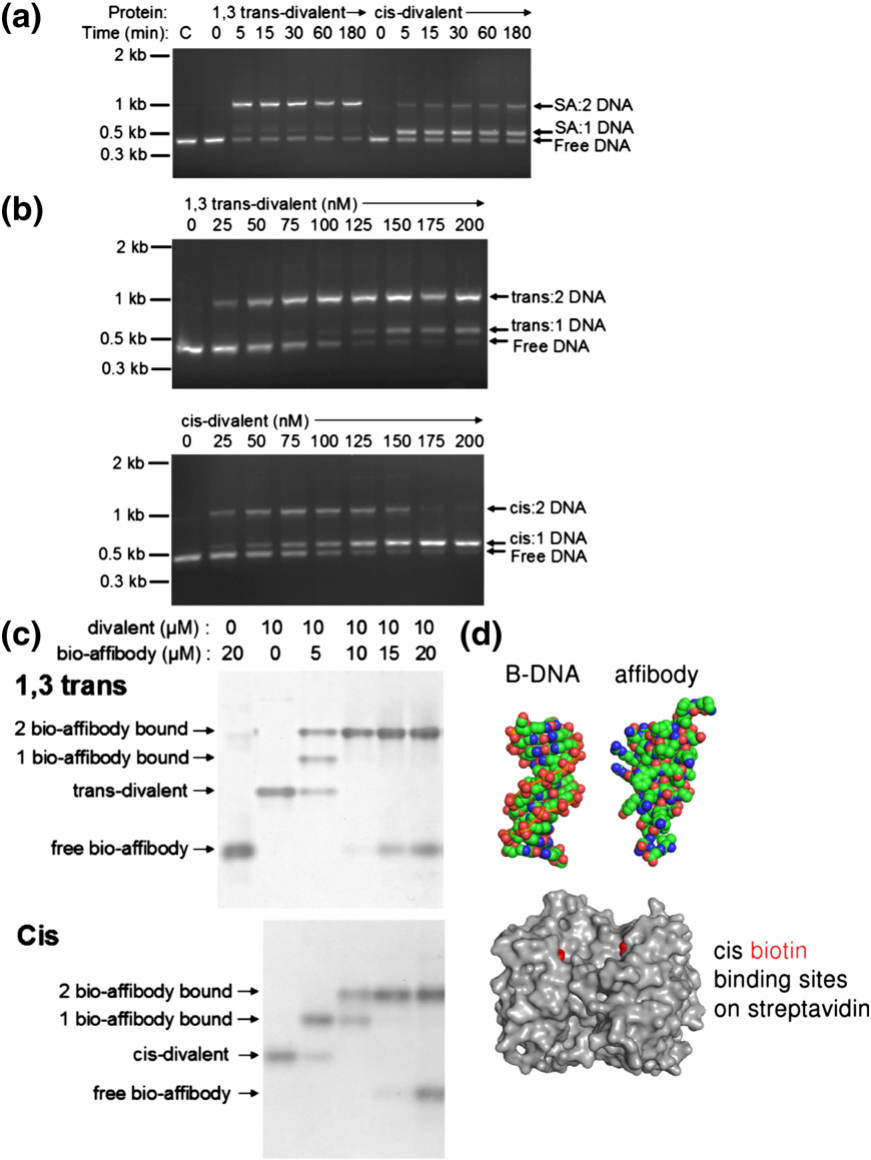
Differential binding of biotinylated molecules by divalent streptavidins. (a) A monobiotinylated PCR product (50 nM) was incubated with 1,3 *trans*- or *cis*-divalent streptavidin (100 nM) for the indicated times at 37 °C and then analyzed by agarose gel electrophoresis, with ethidium bromide visualization of DNA. C is a control of DNA without streptavidin. (b) Titration of the biotinylated PCR product (50 nM) with varying amounts of 1,3 *trans*-divalent (upper panel) or *cis*-divalent (lower panel) streptavidin for 16 h at 25 °C, analyzed by electrophoresis as in (a). (c) Native PAGE of monobiotinylated affibody titrated with 1,3 *trans*-divalent (upper panel) or *cis*-divalent (lower panel) streptavidin, analyzed by Coomassie staining. (d) Comparison of the relative sizes of an affibody (PDB ID: 2KZJ, space-fill), a short segment of B-form DNA (PDB ID: 1BNA, space-fill), and the separation between *cis*-binding sites on streptavidin (PDB ID: 3RY2, protein in gray, biotin in red, Connolly surface).

To characterize further the interaction between the two biotin-binding sites of *cis*-divalent and 1,3 *trans*-divalent streptavidin, we examined the capture of larger biotinylated ligands. Binding of a monobiotinylated 439-bp PCR product was analyzed by a mobility shift in agarose gel electrophoresis (Fig. 7a). The 1,3 *trans*-divalent streptavidin bound two DNA molecules almost immediately. The *cis*-divalent form bound one DNA molecule rapidly, but binding of a second DNA molecule was very slow (Fig. 7a). A similar pattern was observed when titrating the biotinylated DNA with varying concentrations of each divalent streptavidin. While the 1,3 *trans*-divalent streptavidin preferentially bound two molecules of DNA, the *cis*-divalent form bound one or two molecules (Fig. 7b).

We also analyzed binding of a biotinylated protein, avoiding the high charge of polynucleotides. Titration of the divalents with a site-specifically biotinylated affibody [42], analyzed by the mobility shift in native PAGE, yielded a similar pattern (Fig. 7c). With streptavidin binding sites in excess, one or two molecules of the monobiotinylated affibody could bind to the 1,3 *trans*-divalent streptavidin, while for the *cis*-divalent one, only a single affibody bound. However, at higher concentrations of affibody, two molecules of affibody bound to the *cis*-divalent form (Fig. 7c). The sizes of the affibody and DNA double helix relative to streptavidin are illustrated in Fig. 7d.

### Monovalent streptavidin isolated by ion-exchange chromatography allowed specific cellular imaging

Monovalent streptavidin has found diverse uses, such as a highlighter in electron microscopy [43], calibrating energies in protein folding [44], enhancing antibody detection sensitivity [45], and DNA origami-mediated nano-assembly [46]. The clean resolution of monovalent streptavidin SAe1D3 from other valency forms by ion-exchange chromatography (Fig. 2c) is superior to our previous Ni-NTA method, where elution of His_6_-tagged monovalent and divalent forms substantially overlapped [19,29]. Therefore, we validated that the new SAe1D3 form was still effective for cellular imaging and the altered charge did not lead to high non-specific cellular binding. HeLa cells were transfected with ER-resident biotin ligase and acceptor peptide-tagged low-density lipoprotein receptor (LDLR) [29] and then imaged with AlexaFluor 555-labeled SAe1D3. SAe1D3 clearly labeled cells expressing AP-GFP-LDLR but did not label nearby non-expressing cells or cells that were not co-transfected with biotin ligase (Supplementary Data Fig. S4), consistent with specific biotin detection.

Monovalent streptavidin has found diverse uses, such as a highlighter in electron microscopy [43], calibrating energies in protein folding [44], enhancing antibody detection sensitivity [45], and DNA origami-mediated nano-assembly [46]. The clean resolution of monovalent streptavidin SAe1D3 from other valency forms by ion-exchange chromatography (Fig. 2c) is superior to our previous Ni-NTA method, where elution of His_6_-tagged monovalent and divalent forms substantially overlapped [19,29]. Therefore, we validated that the new SAe1D3 form was still effective for cellular imaging and the altered charge did not lead to high non-specific cellular binding. HeLa cells were transfected with ER-resident biotin ligase and acceptor peptide-tagged low-density lipoprotein receptor (LDLR) [29] and then imaged with AlexaFluor 555-labeled SAe1D3. SAe1D3 clearly labeled cells expressing AP-GFP-LDLR but did not label nearby non-expressing cells or cells that were not co-transfected with biotin ligase (Supplementary Data Fig. S4), consistent with specific biotin detection.

## Discussion

Herein, we describe the generation and analysis of tetramers of streptavidin with a defined orientation of twin biotin-binding sites. We were able to engineer *cis*-divalent streptavidin with comparably low biotin off-rate to wild-type streptavidin but 1,3 *trans*-divalent streptavidin showed even better binding stability. Defined divalent streptavidins should act as an efficient and simple bridge for diverse applications in bionanotechnology.

Although new in its application to streptavidin, the separation of multimers by ion-exchange chromatography according to the position of charged tags has been applied previously [47–49]. A similar orientation-dependent separation was observed for wild-type streptavidin tetramers containing two bound biotins because of biotin's charge. However, the biotin occupancy of such streptavidin tetramers changed over hours [27], whereas our defined divalents were stable for at least a week at 37 °C and should be stable indefinitely at − 80 °C. We found high thermostability of both divalent tetramers, with negligible dissociation into monomers until above 60 °C. The introduced mutations have also not reduced biotin-conjugate binding affinity, a problem frequently encountered when engineering streptavidin [14–17].

Despite testing several strategies to isolate 1,4 divalent streptavidins, we could never attain a clean separation. However, we anticipate that the most important thing is having one defined *trans*-divalent streptavidin, since the biotin–biotin separation distance for 1,3 and 1,4 is very close.

The work here also establishes a substantially improved route to monovalent streptavidin. Monovalent streptavidin with an E_6_ tag can be purified on a larger scale compared to the His_6_-tagged monovalent streptavidin, because its elution is well separated from divalent streptavidin. SAe1D3 is also easier to analyze for purity, because it is much better resolved on SDS-PAGE. The E_6_ tag did not impair the use of monovalent streptavidin for specific cellular imaging.

Divalent streptavidins *without* defined orientation have been used for generating monovalent quantum dots [50] and for studying mechanical interactions between MHC class I and T-cell receptors [51], illustrating areas where our defined divalents would improve control in nano-assembly.

Streptavidin and avidin are the most widely used biotin-binding proteins, but recently, several others have been found, including the dimers rhizavidin [52] and shwanavidin [53]. These relatives may find a range of applications, but the natural dimers contain disulfides and provide only one divalent orientation (equivalent to 1,4), and streptavidin appears to have stronger binding to biotin conjugates [53,54]. A further advantage of retaining the whole streptavidin tetramer is the possibility in future work to functionalize Dead subunits, such as by fusion to an antibody fragment [55] or via an exposed cysteine [56], to create a multifunctional hub.

The cooperativity that can exist for biotinylated ligand binding is not well appreciated by the wider community using streptavidin/avidin. For biotin itself, even though streptavidin has a conformational change and increased tetramer stability when biotin binds [18], there is no cooperativity of biotin binding [27]. However, for large biotinylated ligands, there can be substantial *negative* cooperativity in binding to streptavidin/avidin, as shown for biotin metal–ligand complexes [24]. Similarly, the binding of more than two biotinylated DNA molecules to wild-type streptavidin was not readily observed [5,57]. Our defined divalents allowed direct analysis of these ligand–ligand repulsions at *cis* or 1,3 *trans* binding sites. Binding of two large biotinylated ligands was efficient for the 1,3 *trans*-divalent streptavidin. Even with a ligand as small as biotin-4-fluorescein, our structure showed the interactions that would inhibit *cis* binding. For moderate-sized ligands, such as a biotinylated affibody, we found a degree of negative cooperativity for *cis* binding. For a larger and densely charged ligand such as biotinylated DNA, binding in *cis* was both slow and required substantial ligand excess. The two binding sites in the *cis*-divalent streptavidin are 20 Å apart, and the DNA double helix is 20 Å in diameter; hence, binding of the first DNA molecule provides substantial steric and electrostatic hindrance to binding of a second molecule. In particular, the 1,3 *trans*-divalent form should be preferable in generating precise DNA–streptavidin assemblies, as only fully loaded complexes (two DNA molecules per tetramer) were observed, rather than heterogeneous mixtures seen with wild-type streptavidin [5,57].

Numerous chemical (e.g., click chemistry) [58] and biological (e.g., SNAP-tag [59], HaloTag [60,61], SpyTag [62], and coiled coils [63]) approaches have been developed in recent years for stable protein assembly. However, streptavidin is still ubiquitous because of the wide-ranging biotinylated resources available and streptavidin's simple, nearly diffusion-limited, and high-yield binding, enabling “plug and play” construction. Our defined divalent streptavidins should be valuable modules for researchers to contrive novel nano-assemblies using nucleic acids, proteins, sugars, and non-biological building blocks [64].

## Materials and methods

### Constructs

pET21-Core streptavidin (SA) [65] and pET21-Dead streptavidin (D) [19] were used as templates for PCR with KOD Hot Start polymerase (EMD Millipore). For the addition of a C-terminal hexaglutamate tag to core streptavidin to give SAe (GenBank accession number KF378616), 5′-ATACATATGGCTGAAGCTGGTATCACCGGCACCTGG and 5′-CGCAAGCTTTTATTACTCTTCCTCTTCCTCTTCGGAAGCAGCGGACGGTTTAACTTTGG were used. The resulting PCR product was digested with NdeI and HindIII and subcloned into pET21. We inserted a polyaspartate loop into the Dead variant to give Dd (GenBank accession number KF378617) using the Site-directed Ligase-Independent Mutagenesis (SLIM) protocol [66] with 5′-GATGACGATGGTGACGATGACGGTGATGACGATGGAGCTGAATCTAGATACGTTCTGACC, 5′-GCTGAATCTAGATACGTTCTGACC, 5′-TCCATCGTCATCACCGTCATCGTCACCATCGTCATCACCAACAGCGGCTTCGTAGGT, and 5′-ACCAACAGCGGCTTCGTAGGT. pET21b-AP-Affibody was made by inverse PCR [67] from pET21b-ZSPA [68] with an N-terminal acceptor peptide (AP tag, GLNDIFEAQKIEWHE) for BirA-mediated biotinylation [69]. To introduce mutations to bind IGF1R (type 1 insulin-like growth factor receptor) [42], we generated AP-Affibody_IGF1R_ (GenBank accession number KF378618) using the forward primer 5′-AATCGAAAACAGTCTACCGCATTTATTTCTAGCCTTGAAGATGACCCAAGCCAAAGCGCT and the reverse primer 5′-TAGATTCGGTAATGCCAGGATTTCGATTGCAGCATAGAAACCTTCTTTGTTGAATTTGTTGTC.

Biotin ligase with a signal sequence and ER retention sequence (pDisplay-BirA-ER) and pEGFP-AP-GFP-LDLR (human LDLR containing an N-terminal acceptor peptide and GFP) were previously described [70].

All constructs were verified by sequencing. Plasmids for streptavidin constructs are available from Addgene:^2^ D Plasmid 20859, SAe 46367, and Dd 46368.

### Protein expression and purification

SA, SAe, D, and Dd streptavidin variants were expressed in *Escherichia coli* and refolded from inclusion bodies by dilution into phosphate-buffered saline (PBS) as previously described [29]. After refolding, the protein mixtures of SA/Dd or SAe/D were first purified on a 5-mL iminobiotin-Sepharose affinity column (Affiland, S.A.) using 50 mM sodium borate and 300 mM NaCl, pH 11.0, as the binding buffer and 20 mM KH_2_PO_4_, pH 2.2, as the elution buffer, with a 5-mL/min flow rate. The eluate was then exchanged into 20 mM Tris–HCl, pH 8.0, by dialysis and loaded onto a 1-mL Mono-Q column (GE Healthcare). The different tetramers were then isolated using a 100-column-volume (i.e., 100 mL) linear gradient of 0–1 M NaCl, collecting 1-mL fractions with a 1-mL/min flow rate. For larger-scale preparations, a 30-mL Q-Sepharose High-Performance column (GE Healthcare) and a 30-column-volume gradient from 0.15 to 0.4 M NaCl was run. Eluted fractions were concentrated to 5–10 mg/mL using a Vivaspin cutoff 30-kDa centrifugal concentrator (GE Healthcare), dialyzed thrice into PBS, and stored at − 80 °C. All purification steps were performed using an ÄKTA purifier 10 (GE Healthcare). Yield from refolding 1 L of biotin-binding subunit mixed with 1 L of non-binding subunit was approximately 9 mg of the relevant divalent and 12 mg of the monovalent streptavidin.

Monovalent streptavidin (SAe1D3) can also be purified, if preferred, in a gravity-flow column. The mixed refold of SAe and D, prepared in 20 mM Tris–HCl, pH 8.0, as above, is loaded onto 1 mL Q-Sepharose High-Performance resin (GE Healthcare) in a Poly-Prep chromatography column (Bio-Rad) pre-equilibrated with binding buffer of 20 mM Tris–HCl, pH 8.0. The column is washed with 10 mL 20 mM Tris–HCl, pH 8.0, plus 0.15 M NaCl (to wash out D4 and impurities), followed by elution of SAe1D3 with 5 mL 20 mM Tris–HCl, pH 8.0, plus 0.25 M NaCl, collecting 1-mL fractions. The resin is regenerated by washing with 10 mL 20 mM Tris–HCl, pH 8.0, plus 2 M NaCl.

Glutathione *S*-transferase-BirA (the plasmid was a kind gift from Chris O'Callaghan, University of Oxford) was expressed in *E. coli* and purified using glutathione-Sepharose as described previously [71]. AP-Affibody was expressed in *E. coli* and purified using Ni-NTA (Qiagen) [68]. Enzyme-mediated biotinylation was performed as described previously [19].

Protein concentrations were determined from *A*_280_ via ProtParam. Concentrations of all streptavidin forms refer to the concentration of the monomer. p*I* was predicted using ProtParam.

### SDS-PAGE

SDS-PAGE was performed on 10% or 14% polyacrylamide gels, using an XCell SureLock system (Life Technologies). Protein samples (10 μM) for denaturation were mixed with an equal volume of 2 × SDS loading buffer (20% glycerol, 100 mM Tris–HCl, 4% SDS, and 0.2% bromophenol blue, pH 6.8) and heated for 3 min at 95 °C. Non-denatured samples were loaded without boiling, in SDS-free loading buffer. Gels were typically run for 1 h at room temperature at 200 V in running buffer (25 mM Tris–HCl, 192 mM glycine, and 0.1% SDS, pH 8.2).

Biotinylated affibody and streptavidin were incubated at the indicated concentrations in PBS for 16 h at 25 °C, before mixing with an equal volume of 2 × loading buffer (20% glycerol, 100 mM Tris–HCl, and 0.2% bromophenol blue, pH 6.8). Native PAGE was performed on 10% polyacrylamide gels using standard running and loading buffers but without SDS. Gels were stained with InstantBlue (Expedeon) and imaged using a ChemiDoc XRS imager and QuantityOne (version 4.6) software (Bio-Rad).

### Thermostability testing

Samples at 5 μM in PBS were heated at the indicated temperature for 3 min in a DNA Engine® Peltier Thermal Cycler (Bio-Rad). After cooling to room temperature, samples were mixed with an equal volume of 2 × loading buffer (20% glycerol, 100 mM Tris–HCl, and 0.2% bromophenol blue, pH 6.8). SDS-PAGE to discriminate monomers from tetramers was performed on 14% polyacrylamide gels as previously described [36]. Wild-type streptavidin samples heated for 3 min at 95 °C in the presence of SDS were included as controls.

### Divalent streptavidin crystallization and data collection

Apo 1,3 *trans*-divalent streptavidin crystals (space group *P*12_1_1; *a* = 47.04 Å, *b* = 81.59 Å, *c* = 65.12 Å, with two SAe and two D monomers in the asymmetric unit) were obtained from a 1-μL drop of a solution containing 0.5 μL of 740 μM 1,3 *trans*-divalent streptavidin in PBS mixed with 0.5 μL of reservoir solution, 65% v/v MPD, and 0.1 M 2-(*N*-morpholino)ethanesulfonic acid (Mes), pH 6.0. Crystals were obtained by the sitting-drop vapor-diffusion method at 291 K, reached a maximum size after ~ 10 days, and were harvested soon after.

For crystallization of 1,3 *trans*-divalent streptavidin with biotin-4-fluorescein, we added biotin-4-fluorescein to a final concentration of 100 μM along with 75 μM 1,3 *trans*-divalent streptavidin in PBS (0.5 mL final volume). This sample was then concentrated 10-fold using a Vivaspin cutoff 30-kDa centrifugal concentrator. Crystals (space group *P*3_1_21; *a* = 64.41 Å, *b* = 64.41 Å, *c* = 103.13 Å, with one SAe and one D monomer in the asymmetric unit) were obtained from a 1-μL drop of a solution containing 0.5 μL 750 μM 1,3 *trans*-divalent streptavidin mixed with 0.5 μL reservoir solution: 65% v/v MPD and 0.1 M Mes, pH 4.0. Crystals were obtained by the sitting-drop vapor-diffusion method at 277 K, reached a maximum size after ~ 14 days, and were harvested soon after.

Apo *cis*-divalent streptavidin crystals (space group *P*12_1_1; *a* = 46.22 Å, *b* = 84.24 Å, *c* = 58.25 Å, with two SA and two Dd monomers in the asymmetric unit) were obtained from a 0.1-μL drop of a solution containing 113 μM *cis*-divalent streptavidin and 28 μM ALAD-Streptag [11] and a reservoir solution of 2.5% w/v polyethylene glycol 1000, 12.5% w/v polyethylene glycol 3350, 12.5% v/v MPD, 30 mM of ethylene glycol mix (di-ethyleneglycol, tri-ethyleneglycol, tetra-ethyleneglycol, penta-ethyleneglycol), and 0.1 M Mes/imidazole, pH 6.5, corresponding to condition E4 of the Morpheus screen [34]. Crystals appeared after 2 days and were harvested soon after.

Prior to data collection, crystals were mounted and immersed into liquid nitrogen. Crystallographic data were collected at 100 K, using an Oxford Cryosystems 700 series Cryostream on an ADSC Quantum 315 charge-coupled device detector with an oscillation range of 0.5° at beamline iO4 at the Diamond Light Source, Didcot, UK.

### Structure solution and refinement

Data were auto-indexed and integrated by the xia2 program upon collection [72]. The *cis*- and 1,3 *trans*-divalent streptavidin structures were phased by molecular replacement using PDB 3RY1 (apo streptavidin) [35] and further refined using the Phenix [73] suite of programs implemented through a Python graphical user interface [74]. The models were altered to better fit the electron density using Coot [75]. Throughout the refinement of apo 1,3 *trans*-divalent, all data were included from 24.98 Å resolution to the highest limit (1.59 Å), and anisotropic temperature factors were refined. For the 1,3 *trans*-divalent streptavidin with bound biotin-4-fluorescein, all data from 49.06 Å resolution to the highest limit (2.26 Å) and isotropic temperature factors were refined. Similarly, for the *cis*-divalent structure, all data were included from 42.12 Å resolution to the highest limit (1.43 Å), and anisotropic temperature factors were refined. All models were continually evaluated with MolProbity [76].

The refinement statistics and other structure factors for the two structures are shown in Table 1.

Structures were visualized, and images for figures were prepared using PyMOL (Schrödinger, LLC). Electron density maps were visualized and images were prepared (contoured at 1 rmsd) with the CCP4mg program [77]. Predicting the clash between *cis*-bound biotin-4-fluorescein molecules was done by overlaying the biotin-4-fluorescein-bound streptavidin monomer with the adjacent ligand-free monomer using PyMOL.

### Biotin-4-fluorescein off-rate assay

The off-rate of biotin-4-fluorescein (Life Technologies) was measured from the fluorescence increase of biotin-4-fluorescein upon unbinding from streptavidin at 37 °C with competing free biotin (Sigma-Aldrich), as previously described [36]. Samples (180 μL) in a sealed clear 96-well microtiter plate containing 1 μM streptavidin variant in PBS were incubated with 12 nM biotin-4-fluorescein at 37 °C for 1 h. We then added 1 mM biotin (20 μL) and biotin-4-fluorescein release was monitored from the increase in fluorescence (λ_ex_ = 480 nm, λ_em_ = 520 nm) using a SpectraFluor Plus plate reader (Tecan) over time at 37 °C. The percentage of dissociation was calculated as (signal with biotin − signal without biotin)/(signal without quenching − signal without biotin) × 100. The signal without quenching was taken as the biotin-4-fluorescein fluorescence in the absence of streptavidin. One-way ANOVA statistical analysis with Tukey's multiple comparison test for the dissociation rate curves was performed using the Prism program (GraphPad Software).

### Biotin-4-fluorescein titration

To 20 nM streptavidin variant in PBS was added 0 to 15 nM biotin-4-fluorescein. Samples were incubated for 1 h at 37 °C in PBS. Subsequently, the fluorescence was measured (λ_ex_ = 485 nm, λ_em_ = 523 nm) using a SpectraMax M5 plate reader (Molecular Devices).

### Biotinylated DNA gel shift

A monobiotinylated 439-bp PCR product was prepared using Taq polymerase with primer Fts1 and terminally biotinylated primer bioFts3 (5′ biotin triethylene glycol linker), as previously described [36]. Streptavidin variant (100 nM) was incubated with 50 nM monobiotinylated PCR product in electrophoretic mobility shift assay buffer (20 mM Hepes, pH 7.9, 1 mM reduced dithiothreitol, 0.1 mM ethylenediaminetetraacetic acid, 50 mM KCl, 5% glycerol, and 0.2 mg/mL bovine serum albumin) for the indicated time at 37 °C, mixed with 2 × loading buffer [10% glycerol, 2 mg/mL Orange G (Sigma-Aldrich)], and run on a 1.5% agarose gel containing ethidium bromide with TAE (40 mM Tris–HCl, 20 mM acetic acid, and 1 mM ethylenediaminetetraacetic acid, pH 8.3) for 1 h at 130 V. Gels were imaged under UV using a ChemiDoc XRS imager and QuantityOne (version 4.6) software.

For Fig. 7b, the indicated concentration of the streptavidin variant was incubated with 50 nM of the monobiotinylated PCR product in electrophoretic mobility shift assay buffer for 16 h at 25 °C, before mixing with loading buffer and agarose electrophoresis as above.

### Dye labeling

SAe1D3 was labeled with AlexaFluor 555 succinimidyl ester (Life Technologies) according to the manufacturer's instructions. After conjugation (10:1 dye:streptavidin monomer), excess dye was first removed by gel filtration on a PD10 column (GE Healthcare) and subsequently by dialysis three times against excess PBS. The number of dye molecules per streptavidin monomer was calculated, following manufacturer's instructions, from *A*_280_ and *A*_555_ to be 1.2.

### Cell culture and receptor labeling

HeLa cells (American Type Culture Collection) were grown in high-glucose Dulbecco's modified Eagle's medium with 10% fetal bovine serum (GE Healthcare), 50 U/mL penicillin, and 50 μg/mL streptomycin (growth medium). HeLa cells were either co-transfected with AP-GFP-LDLR and BirA-ER plasmids or transfected with AP-GFP-LDLR plasmid alone using Lipofectamine 2000 and incubated overnight in growth medium supplemented with 10 μM biotin. Cells were then placed on ice, rinsed with cold PBS-Mg (PBS + 5 mM MgCl_2_), and labeled with 200 nM SAe1D3-AlexaFluor 555 in PBS-Mg with 1% dialyzed bovine serum albumin for 15 min on ice. After three washes with cold PBS-Mg, cells were imaged live.

### Microscopy

Cells were imaged using a wide-field DeltaVision fluorescence microscope (Applied Precision) with a 40 × oil immersion objective and Optovar lens (1.6 ×), using softWoRx 5.0.0 software (Applied Precision). AlexaFluor 555 was imaged with 540DF40 excitation, 600DF50 emission, and a Chroma 84100bs polychroic filter set. Typical exposure times were 0.1–0.5 s. Samples shown in the same figure were imaged, analyzed, and displayed under identical conditions.

### Accession numbers

Coordinates and structure factors have been deposited with PDB ID: 4BX5 for *cis*-divalent streptavidin, PDB ID: 4BX6 for 1,3 *trans*-divalent ligand-free streptavidin, and PDB ID: 4BX7 for 1,3 *trans*-divalent streptavidin bound to biotin-4-fluorescein.

The following are the supplementary data related to this article.

Supplementary material.

Supplementary material 1.

Supplementary material 2.

Supplementary material 3.

Supplementary data to this article can be found online at http://dx.doi.org/10.1016/j.jmb.2013.09.016.

## Figures and Tables

**Table 1:** Data collection, refinement statistics, and structure validation for the 1,3 *trans*-divalent and *cis*-divalent streptavidin structures.

	1,3 *trans*-divalent (4BX6)	*cis*-divalent (4BX5)	1,3 *trans*-divalent with biotin-4-fluorescein (4BX7)
*Data collection*			
Space group	*P*12_1_1	*P*12_1_1	*P*3_1_21
Asymmetric unit	Tetramer	Tetramer	Dimer
Unit cell size (Å)	47.04 × 81.59 × 65.12	46.22 × 84.24 × 58.25	64.41 × 64.41 × 103.13
Unit cell angles (°)	90 × 95.94 × 90	90 × 98.81 × 90	90 × 90 × 120
Completeness (%)	98.00	92.59	99.77
*Refinement*			
Resolution (Å)	1.59–24.98	1.43–42.12	2.26–49.06
No. of reflections	64,074	75,108	12,059
*R*_work_/*R*_free_	0.1640/0.1866	0.1601/0.1873	0.1839/0.2369
No. of atoms			
Protein	3756	3739	1768
Heteroatoms	24	42	54
Water	466	517	78
*B*-factors (mean of all atoms, Å^2^)			
Wilson plot	20.98	14.65	42.43
Mean *B* value	28.40	22.00	45.10
rmsd			
Bond length (Å)	0.011	0.01	0.01
Bond angle (°)	1.28	1.14	0.94
*Structure validation*			
MolProbity clash score	3.65 (97^th^ percentile)	2.71 (98^th^ percentile)	2.29 (100^th^ percentile)
Poor rotamers (%)	2.01	0.57	2.33
Ramachandran outliers (%)	0.43	0.00	0.00
Ramachandran favored (%)	97.02	97.65	97.85
MolProbity score	1.55 (85^th^ percentile)	1.13 (98^th^ percentile)	1.32 (100^th^ percentile)
Residues with bad bonds (%)	0.00	0.00	0.00
Residues with bad angles (%)	0.00	0.00	0.00

## Footnotes

2 www.addgene.org
