# Supplementary material for: Plug-and-Play Pairing via Defined Divalent Streptavidins
Source: J Mol Biol. 2014 Jan 9;426(1):199–214. doi: 10.1016/j.jmb.2013.09.016 (PMC4047826; doi:10.1016/j.jmb.2013.09.016)
Supplement: Supplementary file 1 — Supplementary material. [file mmc1.pdf]

Supplementary Material for

Plug-and-Play Pairing via Defined Divalent Streptavidins

Michael Fairhead[1], Denis Krndija[1,2], Ed D. Lowe[1] and Mark Howarth[1*]

[1]*Department of Biochemistry, University of Oxford, South Parks Road, Oxford, OX1 3QU, UK* [2]*Current address: Institut Curie, 12 rue Lhomond, 75005 Paris, France*

[*]*Corresponding author:* mark.howarth@bioch.ox.ac.uk

(a)

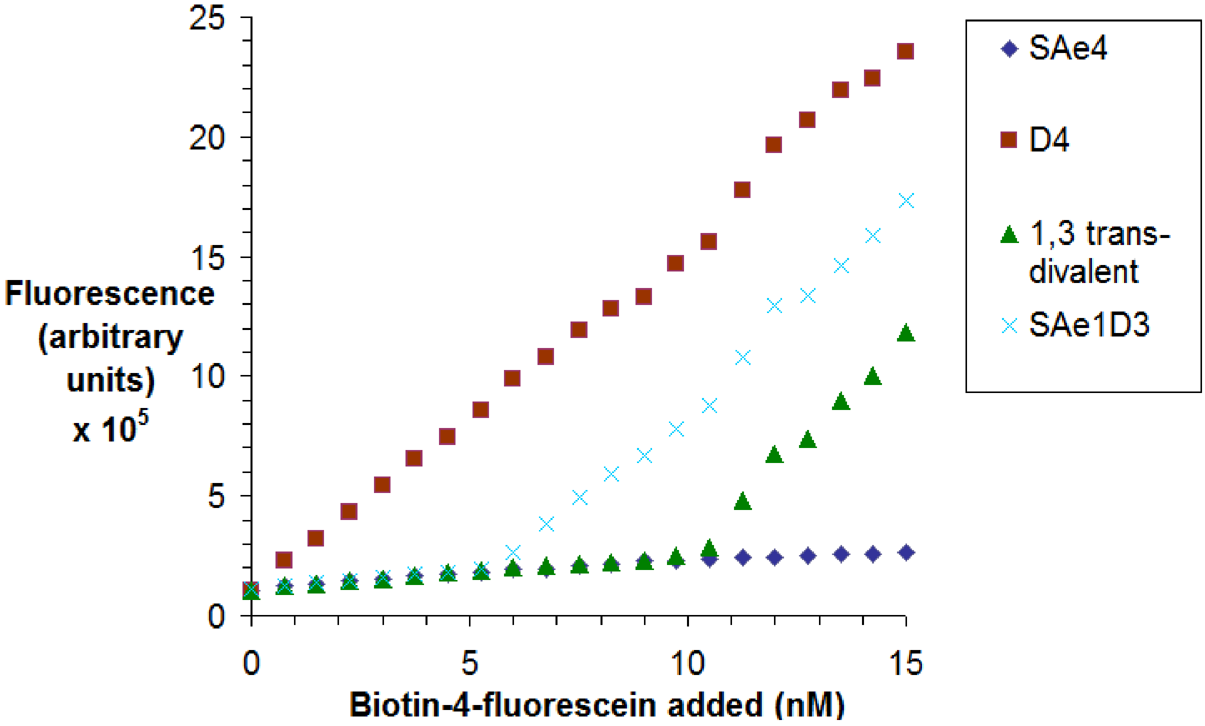

(b)

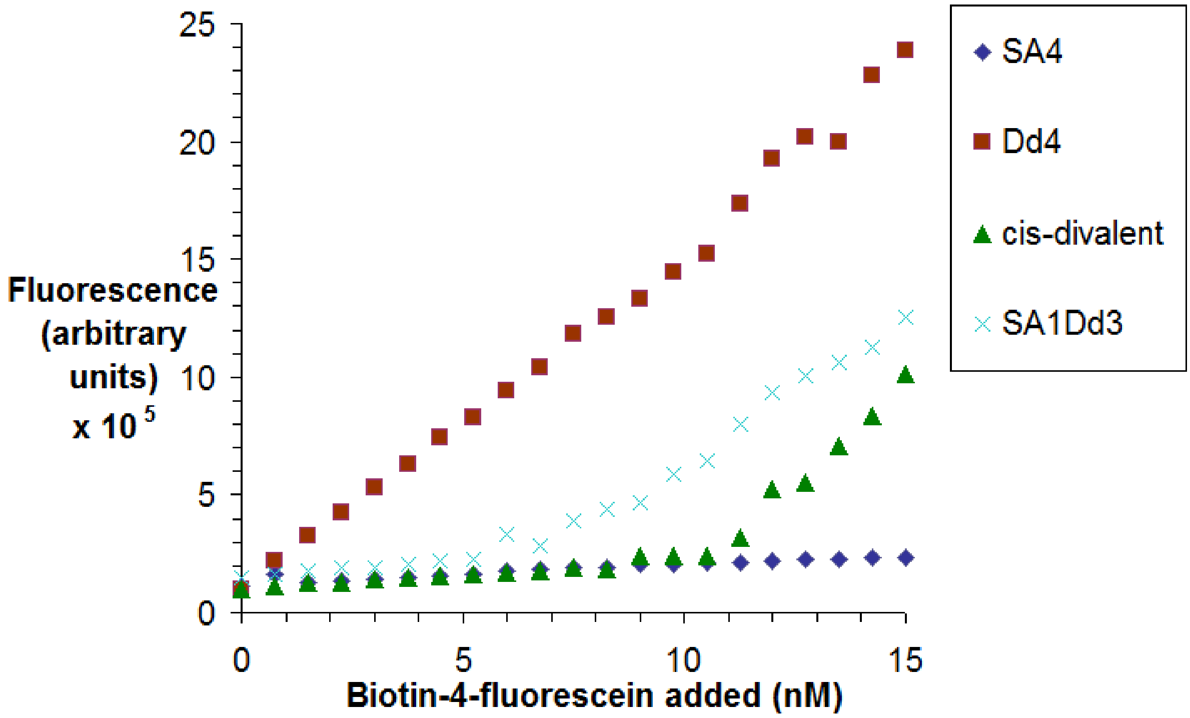

Supplementary Figure S1. Titration of biotin-binding sites. 20 nM of each streptavidin variant (monomer concentration) was incubated with the indicated concentration of biotin-4-fluorescein for 1 h and then fluorescence was measured. As [biotin-4-fluorescein] increases, fluorescence increases slowly at concentrations where all is being bound by streptavidin, but once the biotin-binding sites are saturated, fluorescence increases rapidly.

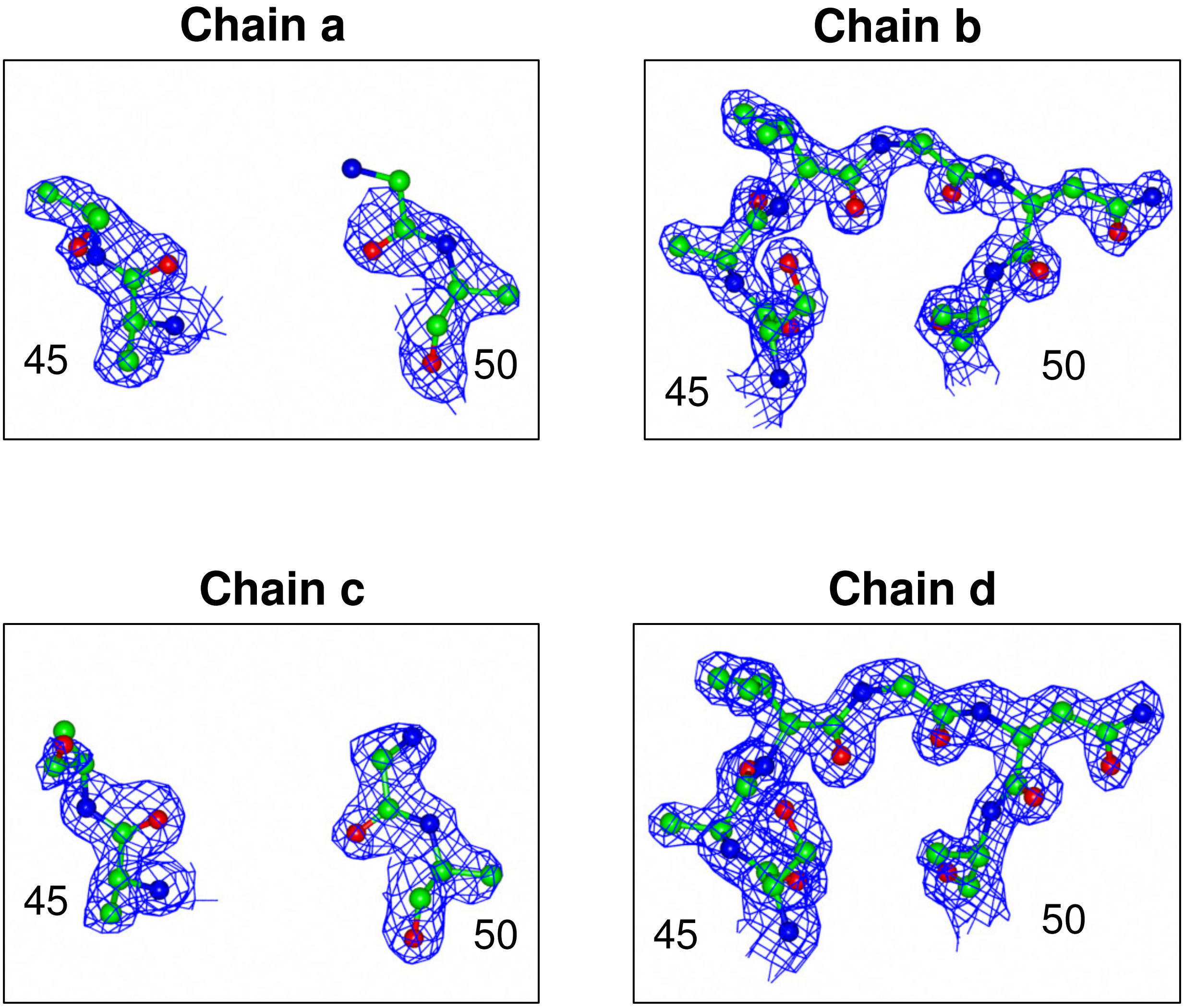

Supplementary Figure S2. Electron density around residues 45-50 in chains a-d of cis-divalent streptavidin ($2F_o$-F_c contoured at 1 rmsd). Residues in stick format are shown surrounded by the observed electron density (blue mesh).

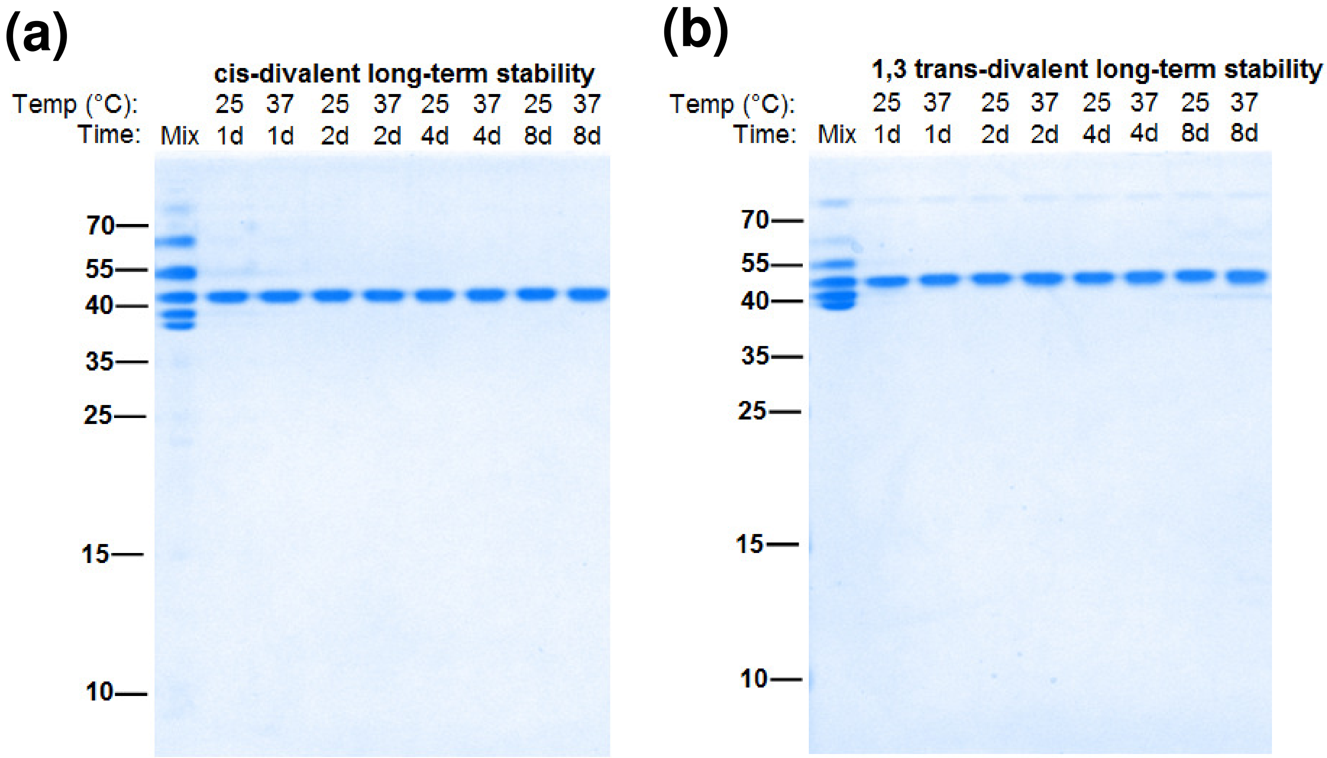

Supplementary Figure S3. Stability over time of cis- and 1,3 trans-divalent streptavidins. 10 μM cis-divalent or 1,3 trans-divalent streptavidin was incubated in PBS at 25°C or 37°C for 1, 2, 4 or 8 days (d). The samples without boiling were then analyzed by SDS-PAGE with Coomassie staining, to check for tetramer rearrangement or dissociation to monomers. The original mixture of unpurified tetramer forms (Mix) provided a reference as to the mobility of possible rearranged products.

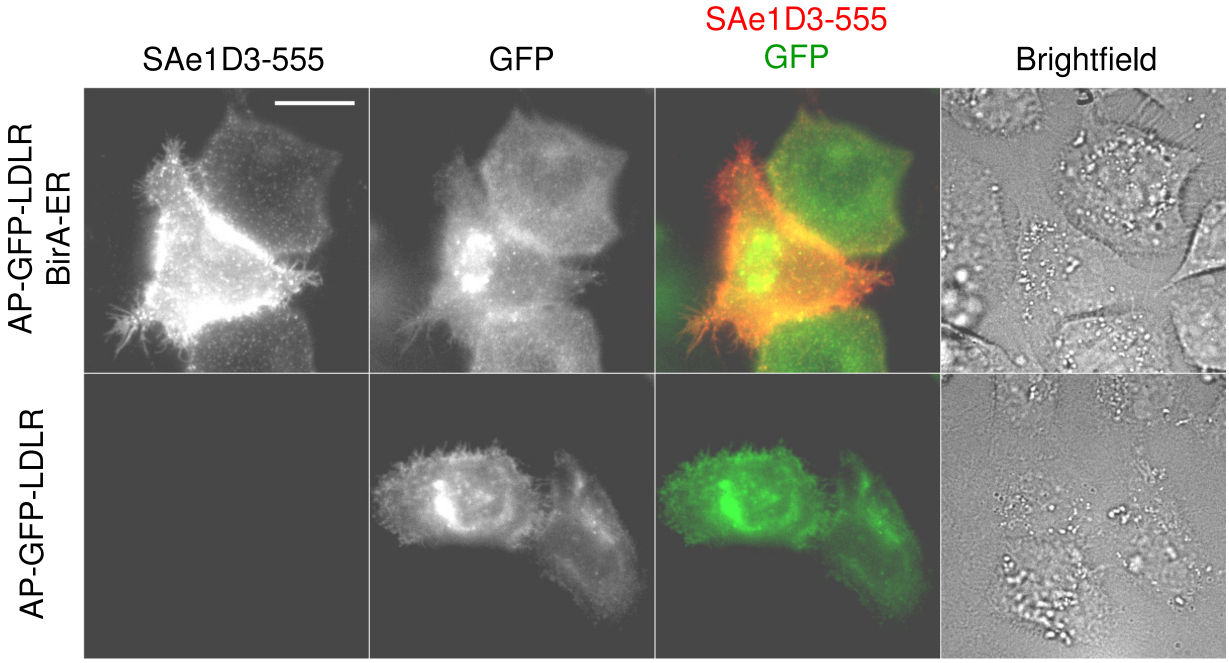

Supplementary Figure S4. Labeling of LDL receptor with SAe1D3. HeLa cells were either co-transfected with AP-GFP-LDLR and BirA-ER (top row) or AP-GFP-LDLR alone (bottom row) and stained with SAe1D3-AlexaFluor 555 before live cell wide-field fluorescence microscopy. Images are shown of SAe1D3-AlexaFluor 555 (left, grayscale), GFP (center left, grayscale), a merge (center right) of SAe1D3-555 (red) and GFP (green), and the brightfield image (right, grayscale). Scale-bar 20 μm.
